# The neurological phoenix: multimodal strategies for brain recovery and prognostication in post-cardiac arrest syndrome—a 2025 clinical framework

**DOI:** 10.3389/fmed.2026.1775538

**Published:** 2026-03-18

**Authors:** Said Kortli, Prashant Nasa

**Affiliations:** 1 Les Hôpitaux de Chartres, Chartres, France; 2 New Cross Hospital, The Royal Wolverhampton NHS Trust, Wolverhampton, United Kingdom

**Keywords:** brain injuries, critical care, heart arrest, post-cardiac arrest syndrome, prognosis

## Abstract

Post-cardiac arrest brain injury remains the leading cause of mortality and morbidity in comatose survivors despite successful resuscitation. This review synthesizes contemporary evidence from the 2025 European Resuscitation Council and European Society of Intensive Care Medicine guidelines, the 2024–2025 International Liaison Committee on Resuscitation recommendations, and recent randomized controlled trials to provide clinicians with a practical framework emphasizing cerebral protection, multimodal monitoring, and reliable prognostication while minimizing premature withdrawal of life-sustaining therapy. Core interventions include targeted oxygenation with peripheral oxygen saturation between 94 and 98% and normocapnia with partial pressure of carbon dioxide between 35 and 45 mm of mercury, individualized perfusion targeting mean arterial pressure of 60–65 mm of mercury, active fever prevention with core temperature maintained at or below 37.5 degrees Celsius for 36–72 h without routine hypothermia, continuous electroencephalography monitoring with treatment of seizures but no prophylactic antiseizure drugs, short-acting sedation enabling neurological assessment, and multimodal neuroprognostication performed at least 72 h post-return of spontaneous circulation requiring concordant predictors across multiple domains. A disciplined multimodal approach utilizing precision in gas exchange and perfusion, rigorous fever prevention, electroencephalography-guided seizure management, and cautious delayed prognostication offers the optimal pathway to meaningful neurological recovery in post-cardiac arrest syndrome.

## Introduction and background

### The burden of post-cardiac arrest syndrome

Cardiac arrest affects over 600,000 individuals annually in the United States, with global incidence ranging from 30 to 97 per 100,000 population (1). Despite advances in resuscitation science, survival rates remain low, with only 9% of out-of-hospital cardiac arrest (OHCA) patients and 23% of in-hospital cardiac arrest (IHCA) patients surviving to discharge (2). Among survivors, hypoxic–ischemic brain injury (HIBI) represents the primary determinant of long-term outcome and accounts for the majority of deaths in successfully resuscitated patients (3).

Post-cardiac arrest syndrome (PCAS) encompasses a complex pathophysiological cascade including brain injury, myocardial dysfunction, and systemic ischemia–reperfusion response leading to multi-organ failure (4). The pathophysiology involves both primary ischemic injury during arrest and secondary reperfusion injury following return of spontaneous circulation (ROSC), characterized by excitotoxicity, inflammation, oxidative stress, and cerebral edema (5, 6).

The 2025 European Resuscitation Council (ERC) and European Society of Intensive Care Medicine (ESICM) guidelines represent a significant evolution in post-resuscitation care, incorporating recent evidence from major trials and providing refined recommendations for temperature management, hemodynamic targets, seizure control, and prognostication (7, 8). This review synthesizes these updates to provide clinicians with an evidence-based framework for optimizing neurocritical care in comatose cardiac arrest survivors.

### Definition of comatose state

For this framework, ‘comatose’ is defined according to Posner et al. (9) and Plum and Posner (10) as a state of unarousable unresponsiveness in which patients do not open their eyes, obey commands, or demonstrate purposeful responses to stimulation. Operationally, this corresponds to a Glasgow Coma Scale (GCS) motor score <6 at ≥72 h post-ROSC, as specified in the 2025 ERC/ESICM guidelines (7, 8).

### Rationale and objectives

The primary objective of this review is to provide a concise, evidence-based intensive care unit (ICU) roadmap to maximize neurological recovery in comatose adults post-cardiac arrest, with emphasis on the 2025 ERC/ESICM recommendations. Secondary objectives include:

- Comparing the 2021 and 2025 guidelines and explaining key changes.
- Appraising evidence for core management pillars including multimodal monitoring, temperature control, hemodynamic targets, and seizure management.
- Highlighting emerging trends and research priorities in neuroprognostication and neuroprotection to improve long-term outcomes.

## Review

### Pathophysiology of hypoxic–ischemic brain injury after cardiac arrest

**Figure 1:**
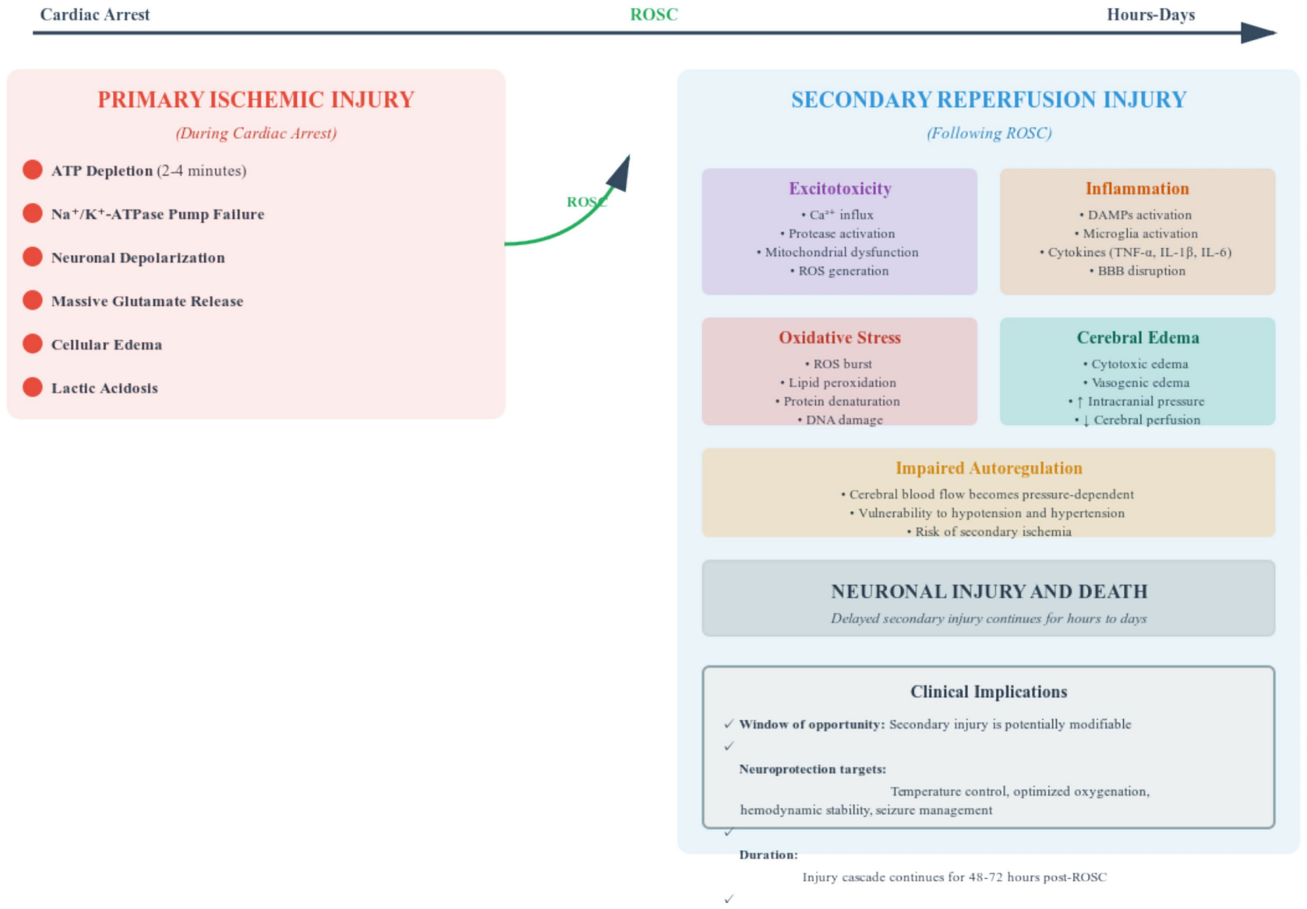
Biphasic pathophysiology of hypoxic–ischemic brain injury after cardiac arrest. This figure illustrates the biphasic nature of hypoxic–ischemic brain injury following cardiac arrest. The primary ischemic phase occurs during cardiac arrest, characterized by adenosine triphosphate depletion within 2–4 min, failure of sodium-potassium-adenosine triphosphatase pumps, neuronal depolarization, massive glutamate release, cellular edema, and lactic acidosis. The secondary reperfusion phase follows return of spontaneous circulation and involves five interconnected mechanisms that continue for hours to days: excitotoxicity with excessive calcium influx and mitochondrial dysfunction; inflammation with damage-associated molecular pattern activation, cytokine release, and blood–brain barrier disruption; oxidative stress with reactive oxygen species burst causing lipid peroxidation and deoxyribonucleic acid damage; cerebral edema in both cytotoxic and vasogenic forms increasing intracranial pressure; and impaired autoregulation rendering cerebral blood flow pressure-dependent. The clinical implications highlighted in this figure emphasize that secondary injury is potentially modifiable through neuroprotective interventions including temperature control, optimized oxygenation, hemodynamic stability, and seizure management, with the injury cascade continuing for 48–72 h post-return of spontaneous circulation necessitating delayed prognostication. This comprehensive understanding of the pathophysiological cascade guides the timing and targets of therapeutic interventions in post- cardiac arrest care. ATP, adenosine triphosphate; ROS, reactive oxygen species; BBB, blood–brain barrier; ROSC, return of spontaneous circulation; TNF-*α*, tumor necrosis factor-alpha; IL-1β, interleukin-1-beta; IL-6, interleukin-6; DNA, deoxyribonucleic acid; GM, grey matter; WM, white matter.

Understanding HIBI pathophysiology is crucial for implementing effective neuroprotective strategies. The injury process occurs in two distinct phases: primary ischemic injury during cardiac arrest and secondary reperfusion injury following ROSC, as illustrated in Figure 1 (5, 11).

### Primary ischemic injury

During cardiac arrest, cessation of cerebral blood flow rapidly depletes neuronal adenosine triphosphate (ATP) stores within 2–4 min, leading to failure of sodium-potassium-adenosine triphosphatase pumps and subsequent neuronal depolarization (5). This triggers massive glutamate release, initiating excitotoxicity cascades. Cellular edema develops from ionic pump failure, while anaerobic metabolism produces lactic acidosis, further impairing cellular function. The duration and severity of this initial anoxic insult critically determine immediate neuronal death and subsequent secondary injury severity (8).

### Secondary reperfusion injury

Paradoxically, restoration of blood flow triggers a second wave of injury through several interconnected mechanisms:

Excitotoxicity occurs through sustained glutamate receptor activation driving excessive calcium influx, activating proteases and lipases while promoting mitochondrial dysfunction and reactive oxygen species (ROS) generation (8, 12).

Inflammation develops as damage-associated molecular patterns activate microglia and recruit peripheral leukocytes, generating pro-inflammatory cytokines including tumor necrosis factor-alpha (TNF-*α*), interleukin-1-beta (IL-1β), and interleukin-6 (IL-6) that disrupt the blood–brain barrier and amplify neuronal injury (13).

Oxidative stress manifests as a reperfusion-induced ROS burst that overwhelms antioxidant defenses, causing lipid peroxidation, protein denaturation, and deoxyribonucleic acid (DNA) damage (14).

Cerebral edema develops in both cytotoxic (intracellular swelling) and vasogenic (blood–brain barrier disruption) forms, increasing intracranial pressure and potentially reducing cerebral perfusion pressure to precipitate secondary ischemia (15).

Impaired autoregulation renders cerebral blood flow pressure-dependent, heightening vulnerability to both hypotension and hypertension (16).

### Initial assessment and management in the ICU

#### Immediate post-resuscitation care and diagnosis

The first hours following ROSC are critical for establishing physiological stability and preventing secondary brain injury. Immediate priorities include securing the airway, optimizing ventilation and oxygenation, and achieving hemodynamic stability with continuous monitoring of vital signs (7, 17).

#### Etiological diagnosis

Establishing the underlying cause of cardiac arrest is critical for neuroprognostication because the etiology directly influences the extent and reversibility of hypoxic–ischemic brain injury. For example, cardiac arrest due to acute coronary occlusion treated with timely revascularization may result in better neurological outcomes compared to prolonged hypoxic arrest from primary respiratory causes. Additionally, identifying non-coronary causes such as intracranial hemorrhage, pulmonary embolism, or aortic dissection is essential as these conditions have distinct pathophysiological mechanisms and prognostic implications that must be considered when interpreting multimodal prognostic tests. Understanding the arrest etiology also guides the interpretation of biomarkers and imaging findings, ensuring that prognostication accurately reflects brain injury severity rather than confounding systemic factors.

Systematic diagnostic workup is essential to identify the underlying cause of cardiac arrest. The 2025 guidelines emphasize early comprehensive imaging, recommending whole-body computed tomography (CT) scanning for patients with OHCA or IHCA of unknown etiology to identify non-coronary causes such as pulmonary embolism, aortic dissection, or intracranial hemorrhage (7, 8, 18).

If there are signs or symptoms pre-arrest suggesting a non-coronary cause—such as headache, seizures, neurological deficits, shortness of breath in patients with known respiratory disease, or abdominal pain—the 2025 guidelines recommend performing a dual-phase whole-body CT scan including head, neck, chest, abdomen, pelvis, and CT pulmonary angiography before or after coronary angiography if indicated (7, 8).

For patients with persistent ST-elevation, immediate coronary angiography is indicated. However, in patients with ROSC after OHCA without ST-elevation on the electrocardiogram (ECG), cardiac catheterization should be delayed unless the clinical context suggests a high likelihood of acute coronary occlusion (7, 8).

**Figure 2:**
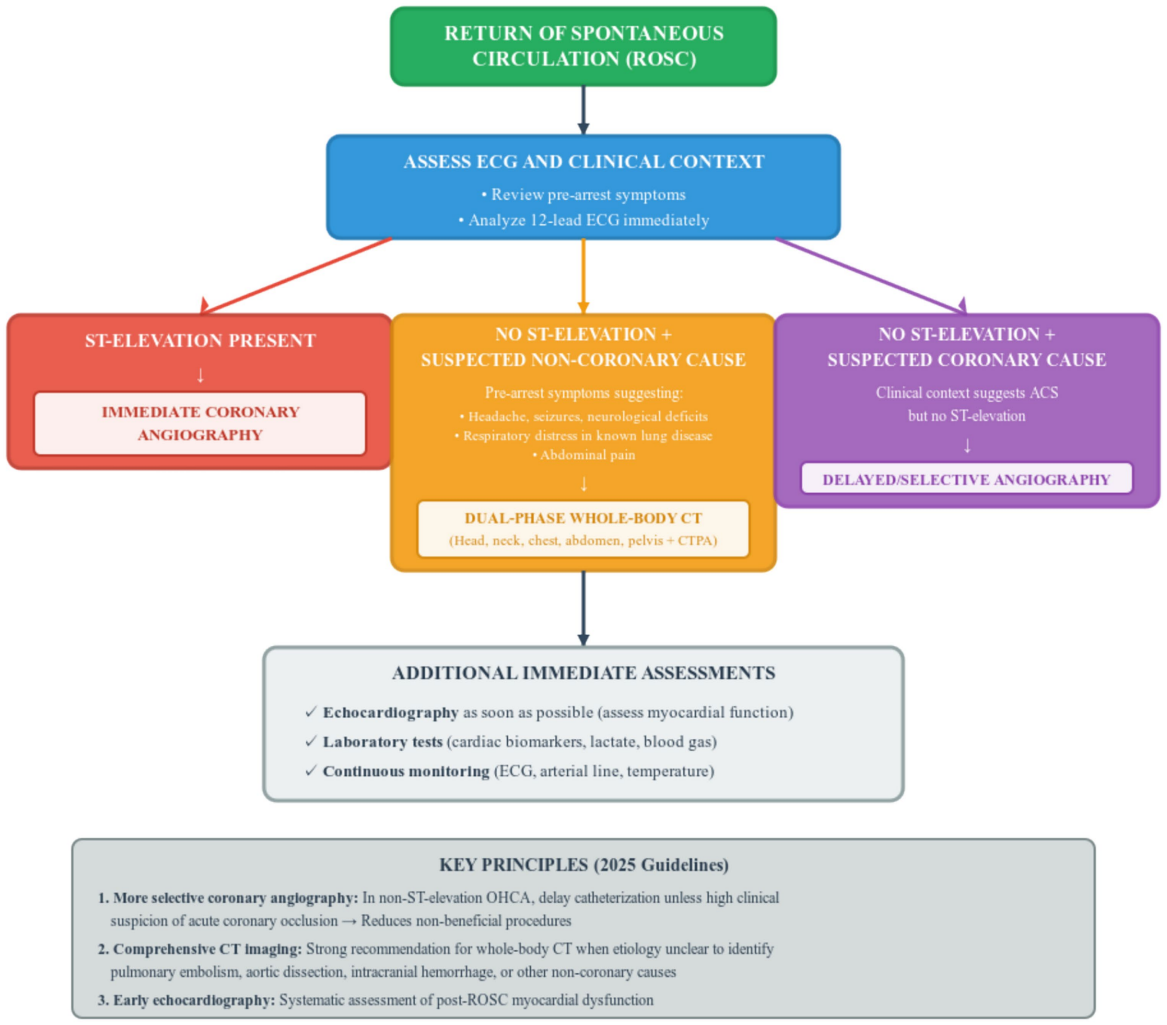
Evidence-based diagnostic algorithm for post-cardiac arrest patients according to 2025 European resuscitation council and European society of intensive care medicine guidelines. This figure presents the diagnostic algorithm for post-cardiac arrest patients emphasizing early identification of the underlying etiology with selective use of invasive and imaging studies based on clinical presentation. Following return of spontaneous circulation, immediate electrocardiogram assessment and clinical context evaluation guide three distinct pathways. The left pathway shows that persistent ST-elevation mandates immediate coronary angiography. The middle pathway demonstrates that patients without ST-elevation but with pre-arrest symptoms suggesting non-coronary causes such as headache, seizures, neurological deficits, respiratory distress in known lung disease, or abdominal pain should undergo dual-phase whole-body computed tomography including head, neck, chest, abdomen, pelvis, and computed tomography pulmonary angiography. The right pathway indicates that patients without ST-elevation but with clinical context suggesting acute coronary syndrome should have delayed or selective coronary angiography rather than immediate catheterization. Additional immediate assessments include echocardiography as soon as possible to evaluate myocardial function, laboratory tests for cardiac biomarkers and lactate, and continuous monitoring with electrocardiogram, arterial line, and temperature. The key principles box at the bottom emphasizes three major 2025 guideline updates: more selective coronary angiography to reduce non-beneficial procedures, comprehensive computed tomography imaging when etiology is unclear to identify pulmonary embolism, aortic dissection, intracranial hemorrhage or other non-coronary causes, and early systematic echocardiography for all patients. ROSC, return of spontaneous circulation; ECG, electrocardiogram; CT, computed tomography; OHCA, out-of- hospital cardiac arrest; CTPA, computed tomography pulmonary angiography; ACS, acute coronary syndrome.

Figure 2 presents the diagnostic algorithm for post-cardiac arrest patients emphasizing early identification of the underlying etiology with selective use of invasive and imaging studies based on clinical presentation.

### Ventilation management

The 2025 guidelines recommend balanced oxygenation targeting peripheral oxygen saturation (SpO_2_) of 94–98%, avoiding both hypoxia and hyperoxia (7, 8). Initial 100% oxygen should be rapidly titrated, as hyperoxia can cause vasoconstriction, reduce cerebral blood flow, and increase oxidative stress (11, 19).

An important update in 2025 emphasizes that pulse oximetry can overestimate the true oxygen saturation in people with darker skin tones, and low-flow states will cause low signal quality (7, 8). This represents a critical safety consideration for accurate oxygen management.

Normocapnia is targeted with partial pressure of carbon dioxide (PaCO_2_) between 35 and 45 mm of mercury (mmHg) or 4.7–6.0 kilopascals (kPa) (7, 8). Hypocapnia causes cerebral vasoconstriction, reducing cerebral blood flow, while hypercapnia leads to vasodilation potentially increasing intracranial pressure in patients with impaired autoregulation (20, 21).

In patients with accidental hypothermia or treated with hypothermia, the 2025 guidelines recommend monitoring PaCO_2_ frequently as hypocapnia may occur (7, 8). Clinicians should use consistently either temperature-corrected or non-temperature-corrected blood gas values.

**Figure 3:**
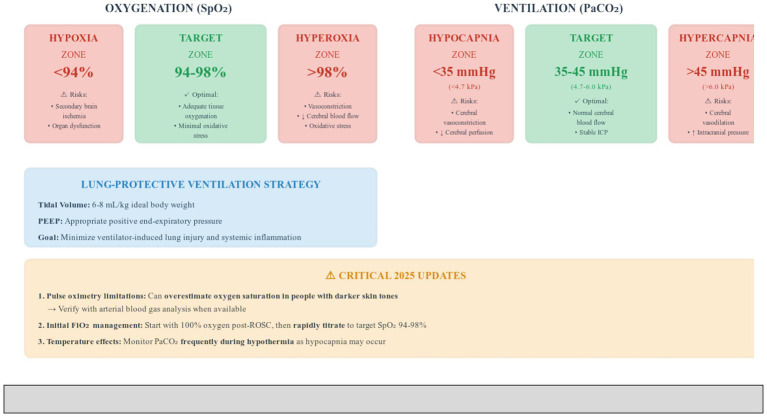
Recommended Oxygenation and Ventilation Targets for Post- Cardiac Arrest Patients with Critical 2025 Updates. This figure demonstrates the recommended physiological targets for oxygenation and ventilation management in post-cardiac arrest patients according to 2025 ERC and ESICM guidelines. The oxygenation section displays three zones: the hypoxia zone with peripheral oxygen saturation below 94% carries risks of secondary brain ischemia and organ dysfunction; the target zone of 94–98% provides optimal tissue oxygenation with minimal oxidative stress; and the hyperoxia zone above 98% risks cerebral vasoconstriction, reduced cerebral blood flow, and increased oxidative stress. The ventilation section similarly shows three zones: the hypocapnia zone with partial pressure of carbon dioxide below 35 mm of mercury or 4.7 kilopascals causes cerebral vasoconstriction and reduced perfusion; the target zone of 35–45 mm of mercury or 4.7–6.0 kilopascals maintains normal cerebral blood flow and stable intracranial pressure; and the hypercapnia zone above 45 mm of mercury or 6.0 kilopascals leads to cerebral vasodilation and potentially increased intracranial pressure. The lung-protective ventilation strategy box emphasizes tidal volumes of 6–8 milliliters per kilogram ideal body weight with appropriate positive end-expiratory pressure to minimize ventilator-induced lung injury and systemic inflammation. SpO_2_, peripheral oxygen saturation; PaCO_2_, partial pressure of carbon dioxide; mmHg, millimeters of mercury; kPa, kilopascals; PEEP, positive end-expiratory pressure; FiO_2_, fraction of inspired oxygen; ICP, intracranial pressure; ABG, arterial blood gas.

Lung-protective ventilation with tidal volumes of 6–8 milliliters per kilogram ideal body weight with appropriate positive end-expiratory pressure (PEEP) minimizes ventilator-induced lung injury and systemic inflammation (7, 8, 22) (Figure 3).

### Core neurocritical care strategies

#### Hemodynamic management for cerebral protection

Maintaining adequate cerebral perfusion is fundamental to preventing secondary brain injury. Post-cardiac arrest myocardial dysfunction and systemic vasodilation frequently cause hypotension, compromising cerebral blood flow (23).

The 2025 guidelines specify a mean arterial pressure (MAP) target of 60–65 mmHg, refined from the 2021 recommendation of MAP at least 65 mmHg (7, 8). This recommendation is based on a 2023 systematic review analyzing over 1,000 patients, which found no association between higher MAP targets greater than 71 mmHg and improved survival, functional outcomes, or reduced acute kidney injury (7, 8, 24).

**Figure 4:**
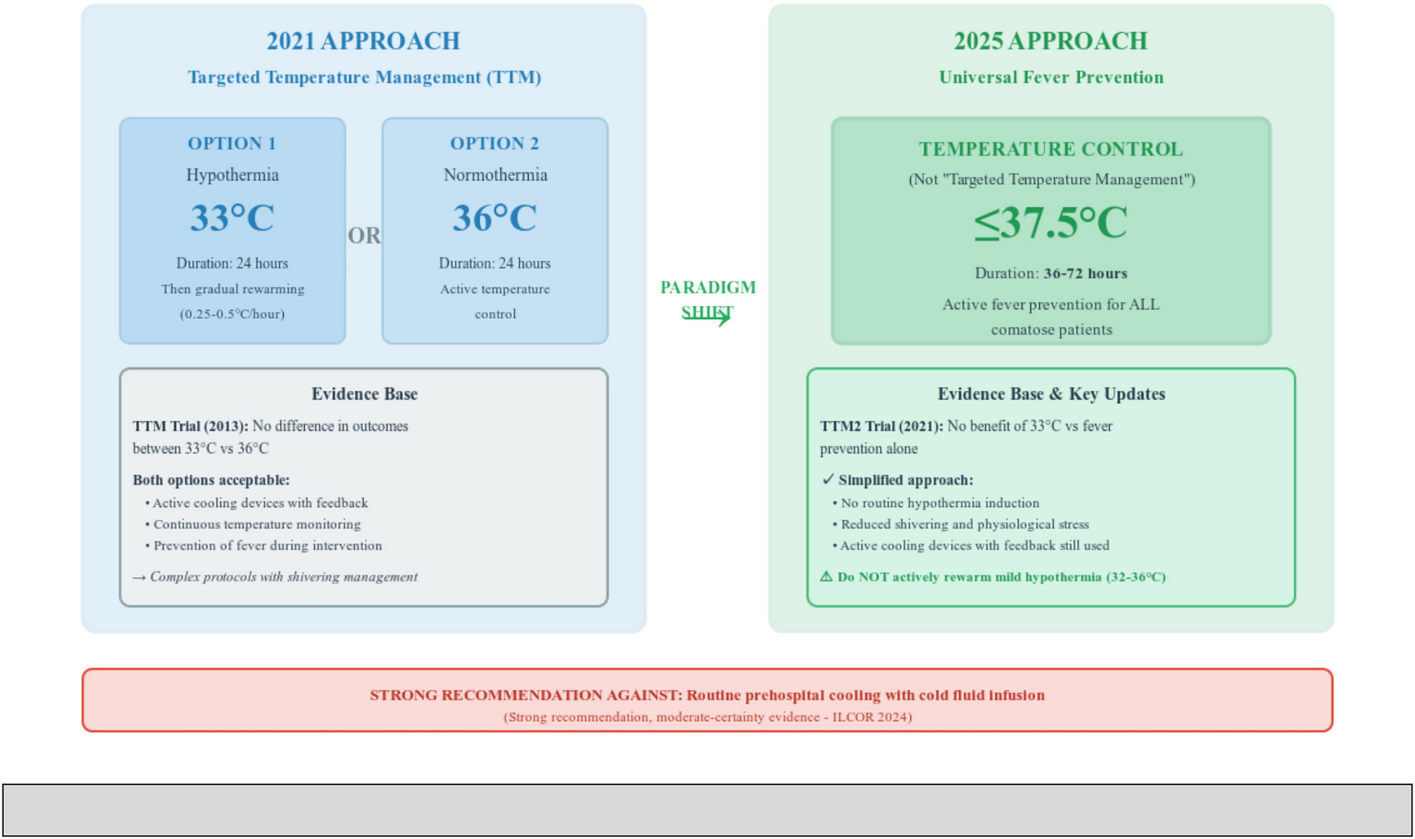
Paradigm Shift in Temperature Management Strategy from Targeted Temperature Management to Universal Fever Prevention. This figure illustrates the major paradigm shift in temperature management for comatose post-cardiac arrest patients between 2021 and 2025 European Resuscitation Council and European Society of Intensive Care Medicine guidelines. The left panel shows the 2021 approach of targeted temperature management offering two options: hypothermia at 33 degrees Celsius for 24 h followed by gradual rewarming at 0.25–0.5 degrees Celsius per hour, or normothermia at 36 degrees Celsius for 24 h with active temperature control. This approach was based on the Targeted Temperature Management trial from 2013 showing no difference in outcomes between the two temperature targets, though both options required complex protocols with shivering management and active cooling devices with feedback systems. The right panel demonstrates the simplified 2025 approach of universal fever prevention targeting core body temperature at or below 37.5 degrees Celsius for 36–72 h for all comatose patients, with no routine hypothermia induction and preferred terminology of temperature control rather than targeted temperature management. This change is based on the Targeted Temperature Management 2 trial from 2021 showing no benefit of 33 degrees Celsius compared to fever prevention alone. The 2025 approach offers a simplified protocol with reduced shivering and physiological stress while still using active cooling devices with feedback when needed. An important caution emphasizes not actively rewarming patients with mild hypothermia between 32 and 36 degrees Celsius. The bottom recommendation box highlights the strong recommendation against routine prehospital cooling with cold fluid infusion based on moderate-certainty evidence from International Liaison Committee on Resuscitation 2024 guidelines. TTM, targeted temperature management; ROSC, return of spontaneous circulation.

However, individualized targets may be appropriate based on baseline blood pressure, comorbidities, and end-organ perfusion markers. A higher MAP target might be appropriate in patients with chronic hypertension or those with persistent peripheral hypoperfusion despite MAP of 60–65 mmHg, such as oliguria or persistently elevated lactate levels (7, 8) (Figure 4).

While the International Liaison Committee on Resuscitation (ILCOR) found insufficient evidence to recommend a specific vasopressor, noradrenaline is generally preferred as first-line therapy due to its potent vasoconstrictive effects with minimal impact on cardiac output (7, 8, 25). Limited evidence from one small randomized trial showed identical 30-day mortality with noradrenaline versus adrenaline at 90% in both groups (7, 8, 26). In settings where noradrenaline is not available such as prehospital environments, the use of adrenaline as an infusion or as small boluses may be an accepted approach according to the 2025 guidelines (7, 8).

Continuous arterial blood pressure monitoring with frequent arterial blood gas analysis is essential. Echocardiography should be performed as soon as possible in all patients to detect any underlying cardiac pathology and quantify the degree of myocardial dysfunction (7, 8, 27).

### Temperature control

Temperature management has undergone significant evolution in the 2025 guidelines, with current evidence supporting universal fever prevention rather than targeted hypothermia for all patients.

The guidelines now strongly recommend actively preventing fever by targeting core body temperature at or below 37.5 degrees Celsius for all comatose patients (7, 8). This represents a shift from previous hypothermia recommendations, based on evidence from trials such as the Targeted Temperature Management 2 (TTM2) trial demonstrating no additional benefit of targeted hypothermia at 33 degrees Celsius compared to targeted normothermia (7, 8, 28). The term “temperature control” is now preferred over “targeted temperature management” (7, 8).

The 2024 ILCOR guidelines, adopted in the 2025 ERC/ESICM guidelines, suggest:

- Actively preventing fever by targeting temperature at or below 37.5 degrees Celsius (weak recommendation with low-certainty evidence).
- Against routine prehospital cooling with cold fluid infusion (strong recommendation with moderate-certainty evidence).
- Using temperature control devices with feedback systems when cooling is employed (7, 8, 25).

Fever prevention should continue for 36–72 h using active cooling devices with continuous temperature monitoring and feedback systems (7, 8). Surface or endovascular cooling techniques are preferred when temperature control is indicated. Importantly, comatose patients with mild hypothermia between 32 and 36 degrees Celsius after ROSC should not be actively warmed to achieve normothermia (7, 8).

### Seizure management and neurological monitoring

Post-cardiac arrest seizures occur in 20–30% of comatose survivors, typically indicating severe hypoxic–ischemic encephalopathy (29). Both convulsive and non-convulsive seizures can worsen secondary brain injury by increasing metabolic demand.

Myoclonus represents the predominant seizure phenotype, characterized by sudden, brief, shock-like muscle contractions that may be generalized, focal, or multifocal (30). Onset typically occurs within 24–48 h post-arrest. Lance-Adams syndrome, emerging after consciousness recovery, predominantly affects extremities and is triggered by voluntary movement (31).

Continuous or routine electroencephalography (EEG) is recommended for at least 24–48 h to detect non-convulsive status epilepticus, which may only be apparent on electroencephalography (7, 8, 32). Standardized criteria exist for diagnosing electrographic seizures using the American Clinical Neurophysiology Society (ACNS) terminology (7, 8, 33). The 2025 guidelines emphasize recording EEG from day 1 after ROSC to predict outcome and detect seizure activity in comatose patients, with either routine EEG or continuous EEG monitoring being acceptable (7, 8).

The 2024 ILCOR recommendations, adopted by ERC/ESICM, suggest against prophylactic antiseizure medications (weak recommendation with very low-certainty evidence) but recommend treating clinically apparent and EEG-detected seizures (good practice statement) (7, 8, 25). First-line agents include levetiracetam or sodium valproate, with drug selection based on comorbidities and potential interactions (7, 8, 34).

For patients with myoclonus and benign EEG background, the 2025 guidelines recommend attempting a wake-up trial days after arrest (7, 8). The guidelines also suggest recording the EEG in the presence of myoclonic jerks to detect any associated epileptiform activity or to identify EEG signs, such as background reactivity or continuity, suggesting a potential for neurological recovery (7, 8).

### General intensive care and sedation

While the primary focus of post-cardiac arrest care is cerebral protection and prognostication, comprehensive general ICU management is essential for optimizing neurological recovery. Secondary brain injury can be exacerbated by systemic complications including gastrointestinal bleeding from stress ulceration (especially in patients receiving antiplatelet or anticoagulant therapy), hyperglycemia or hypoglycemia causing metabolic derangements, malnutrition impairing neuronal repair mechanisms, and infections particularly aspiration pneumonia triggering systemic inflammation and fever. Each of these complications can confound neurological assessment and worsen secondary brain injury. Therefore, meticulous attention to these general ICU care elements is not merely supportive but directly impacts the accuracy of prognostication and the potential for meaningful neurological recovery.

Short-acting sedatives such as propofol or remifentanil are recommended to facilitate neurological assessment and wake-up trials (7, 8). Limited evidence suggests propofol-remifentanil combinations may reduce time to awakening, though with increased vasopressor requirements (7, 8, 35). The 2025 guidelines emphasize using short-acting sedative agents and daily sedation holds when treating post-cardiac arrest patients receiving mechanical ventilation, which may enable earlier clinical examination that is less confounded by sedation when assessing neurological recovery (7, 8).

While the 2025 guidelines emphasize short-acting sedation and daily sedation holds to enable neurological assessment, no standardized protocol for sedation interruption timing, duration, or monitoring currently exists. This represents an important implementation gap. Future research should establish best-practice protocols to operationalize consistent sedation holds, minimize assessment confounding, and optimize prognostic accuracy.

Routine use of neuromuscular blocking agents is discouraged unless clinically indicated for severe acute respiratory distress syndrome (ARDS), refractory status epilepticus, or prevention of shivering during temperature management, as they can mask seizures and hinder neurological assessment (7, 8, 36). The 2025 guidelines explicitly state that systematic use of neuromuscular blocking drugs in comatose post-cardiac arrest patients is not recommended (7, 8). In patients with critical hypoxemia and ARDS following cardiac arrest, the use of a neuromuscular blocker may be considered (7, 8).

Comprehensive ICU management includes:

Stress ulcer prophylaxis is especially important given the high incidence of upper gastrointestinal ulceration in post-cardiac arrest patients and the use of anticoagulant and antiplatelet drugs (7, 8).

Individualized anticoagulation should be based on general ICU recommendations (7, 8).

Strict glycemic control using standard glucose management protocols is recommended (7, 8).

Early enteral nutrition should be initiated by starting gastric feeding at low rates as trophic feeding and increasing as tolerated (7, 8).

Infection prevention strategies should be implemented (37). The 2025 guidelines recommend against routine use of prophylactic antibiotics in patients following ROSC but suggest maintaining a low threshold for giving antibiotics when there is any clinical suspicion of pneumonia (7, 8).

Pressure injury prevention: Patients are at particularly high risk for pressure injuries given prolonged immobility, compromised tissue perfusion, neurological impairment, incontinence, and nutritional vulnerability. Regular repositioning, specialized support surfaces, and systematic skin integrity assessments are essential to prevent secondary complications that may compromise rehabilitation potential.

### Neurological prognostication and long-term outcomes

Accurate prognostication is crucial for guiding clinical decisions and avoiding premature withdrawal of life-sustaining therapy (WLST). The 2025 guidelines emphasize a multimodal approach performed at least 72 h post-ROSC after excluding confounding factors (7, 8).

### Outcome definitions

The 2025 guidelines define:

Poor neurological outcome as death or severe disability (Cerebral Performance Category [CPC] 3–5 or modified Rankin Scale [mRS] 4–6), typically corresponding to patients who remain unconscious or severely dependent at hospital discharge or 6-month follow-up.

Favorable outcome as functional independence (CPC 1–2 or mRS 0–3), typically with return of consciousness, meaningful communication, and ability to perform activities of daily living with minimal or no assistance.

The Glasgow Outcome Scale Extended (GOSE) provides additional granularity for functional recovery assessment (38, 39).

### Multimodal assessment framework

#### Clinical examination

Clinical examination remains a cornerstone of prognostication. The 2025 ILCOR recommendations, adopted by ERC/ESICM, suggest:

- Using pupillary light reflex at 72 h or later for predicting outcomes (weak recommendation with very-low-certainty evidence) (7, 8).
- Using a pupillometer when available to determine if the pupillary light reflex is absent (7, 8).
- Using bilateral absence of corneal reflex at 72 h or later for predicting poor neurological outcome (weak recommendation with very low-certainty evidence) (7, 8).
- The presence of myoclonus or status myoclonus within 7 days, in combination with other tests, may be used for predicting poor outcome (weak recommendation with very low-certainty evidence) (7, 8).

Status myoclonus is defined as continuous and generalized myoclonus persisting for 30 min or more within 72 h after ROSC (7, 8). Recording EEG in the presence of myoclonic jerks is recommended to detect any associated epileptiform activity (7, 8, 40).

Clinical examination is prone to interference from sedatives, opioids, or muscle relaxants, and potential confounding from residual sedation should always be considered and excluded (7, 8).

Advanced Behavioral Assessment: While the GCS motor score remains the primary threshold for prognostication (motor <6), emerging evidence emphasizes the value of specialized behavioral assessment tools beyond the GCS. The Coma Recovery Scale-Revised (CRS-R) and other validated Disorders of Consciousness (DoC) assessments can detect subtle signs of consciousness such as visual tracking, command-following, or emotional expression that the GCS may miss.

The American Academy of Neurology (41) and European Academy of Neurology (42) provide evidence-based recommendations for systematic consciousness assessment to distinguish vegetative state (unresponsive wakefulness syndrome) from minimally conscious states (42, 43). Incorporation of these structured assessments into multimodal prognostication may reduce misdiagnosis and guide rehabilitation planning, particularly when clinical examination yields indeterminate findings.

Important Limitation: Clinicians should recognize the well-documented limitations of the GCS, particularly in detecting subtle signs of consciousness across the spectrum of DoC (44). The GCS was originally designed for trauma assessment, not nuanced consciousness evaluation in hypoxic–ischemic brain injury. Combining GCS with specialized DoC assessments (CRS-R) may improve diagnostic and prognostic accuracy.

#### Neurophysiological assessment

The 2025 guidelines provide refined EEG criteria:

EEG patterns: Suppression or burst-suppression on EEG, defined as highly malignant patterns including suppressed background with or without periodic discharges or burst-suppression according to ACNS, being accurate indicators of poor prognosis when assessed after 24 h from ROSC (7, 8).

Important change: The guidelines recommend against using absence of EEG background reactivity alone to predict poor outcome (weak recommendation with very low-certainty evidence) (7, 8). This represents a significant change from previous practice.

SSEPs: The bilateral absence of somatosensory evoked cortical N20 potentials indicates poor prognosis after cardiac arrest (7, 8). Somatosensory evoked potentials (SSEPs) can be recorded at 24 h or later from ROSC, which changed from previous recommendations (7, 8). The guidelines recommend always considering the use of a neuromuscular blocking drug when performing SSEPs (7, 8).

#### Biomarker assessment

The 2025 guidelines maintain neuron-specific enolase (NSE) as the recommended biomarker, suggesting the use of serial measurements of NSE to predict outcome after cardiac arrest (7, 8).

- NSE greater than 60 micrograms per liter at 48 h and/or 72 h after ROSC indicates poor prognosis (7, 8).
- Increasing NSE values between 24 and 48 h or between 24/48 and 72 h further support a likely poor outcome (7, 8).
- The guidelines recommend performing serial NSE samples at 24, 48, and 72 h after ROSC to detect NSE trends and minimize confounding from occasional hemolysis (7, 8).

The 2025 guidelines do not recommend using neurofilament light chain (NfL), S-100B protein, glial fibrillary acidic protein (GFAP), or serum tau protein for prognostication due to absence of consistent thresholds and mostly research-only assays (7, 8).

### Brain imaging

The guidelines recommend using brain imaging studies to predict poor neurological outcome after cardiac arrest, ensuring that images are evaluated by someone with specific experience in these studies (7, 8).

CT findings: The 2025 guidelines recommend using the presence of generalized brain edema, manifested by a marked reduction of the grey matter to white matter ratio on brain CT, to predict poor neurological outcome (7, 8). The guidelines recommend repeating the brain CT if the patient is unconscious at the time of prognostication, which occurs 72–96 h after ROSC, and the first brain CT does not show signs of HIBI (7, 8).

Where specialist neuroradiology expertise is unavailable, the guidelines suggest considering telemedicine consultation for brain imaging interpretation (7, 8).

MRI findings: The guidelines recommend using extensive diffusion restriction on brain MRI at 2–7 days after ROSC to predict poor neurological outcome (7, 8). Brain imaging should only be used in centers with specific experience (7, 8).

### Prognostication algorithm

The refined 2025 algorithm represents a significant evolution from 2021, with several critical updates. The 2025 guidelines recommend considering neurological prognostication in patients who are not awake and obeying commands, defined as Glasgow Coma Scale motor score less than 6, at 72 h or later after ROSC (7, 8). This represents a change from the 2021 threshold of motor response at or below 3.

Core principles include:

1 Starting prognostication assessment at 72 h or later from ROSC (7, 8).
2 Excluding major confounders including analgesics, sedation, neuromuscular blockade, hypothermia, severe hypotension, hypoglycemia, sepsis, and metabolic and respiratory derangements (7, 8).
3 Requiring a multimodal approach as no single predictor is 100% accurate (7, 8).
4 Requiring two concordant predictors minimum (7, 8).

Poor outcome prediction: In an unconscious patient at 72 h from ROSC, in the absence of confounders, poor outcome is likely when two or more of the following predictors are present:

- No pupillary and corneal reflexes at 72 h (with use of pupillometer when available).
- Bilaterally absent N20 SSEP wave at 24 h or later.
- Highly malignant EEG at >24 h (defined as suppression or burst-suppression off sedation).
- NSE > 60 μg/L at 48 and/or 72 h, or increasing NSE between timepoints.
- Status myoclonus at or before 72 h (continuous and generalized myoclonus for ≥30 min).
- Diffuse and extensive anoxic injury on brain CT or MRI (7, 8).

Favorable outcome prediction: The 2025 guidelines place significant new emphasis on identifying patients with potential for recovery. When none of the criteria for poor outcome are present, the guidelines recommend assessing for signs of potential recovery including:

- Glasgow Coma Scale motor score of 4 or 5 at 72–96 h after ROSC.
- Normal blood values of NSE < 17 μg/L at 24–72 h after ROSC.
- Continuous background without discharges on EEG within 72 h from ROSC.
- Absent diffusion restriction in cortex or deep grey matter on MRI on days 2–7 after ROSC (7, 8).

In patients with two or more concordant favorable signs and no signs of poor outcome, the neurological recovery rate was greater than 80% (7, 8).

**Figure 5:**
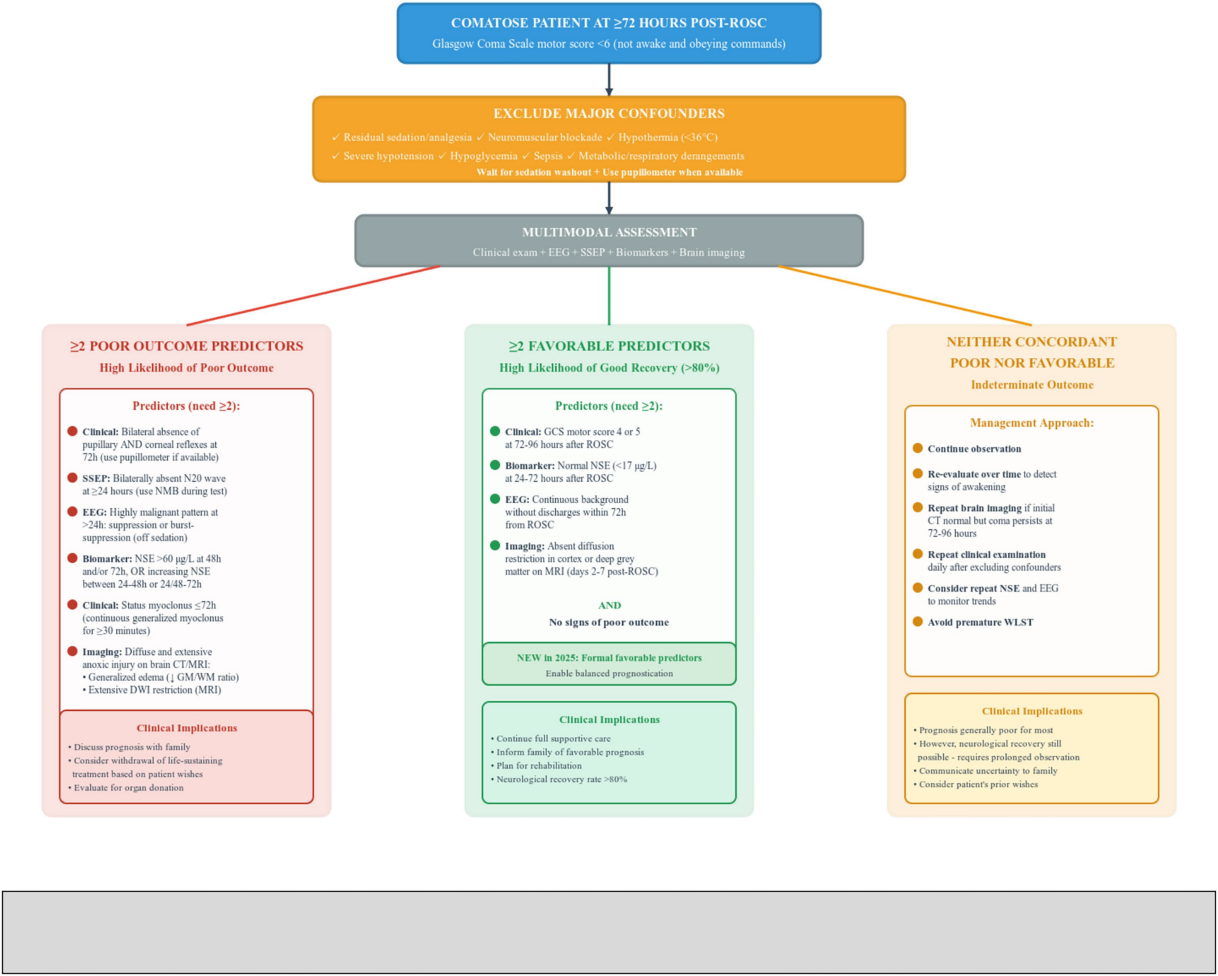
Comprehensive Multimodal Neuroprognostication Algorithm for Comatose Post-Cardiac Arrest Patients at 72 h or Later Following Return of Spontaneous Circulation. This figure presents the 2025 multimodal neuroprognostication algorithm for comatose post-cardiac arrest patients at 72 h or later following return of spontaneous circulation, defined as Glasgow Coma Scale motor score less than 6. After excluding confounders including sedation, neuromuscular blockade, hypothermia, hypotension, and metabolic derangements, three pathways emerge. The left pathway indicates poor outcome when two or more predictors are present: bilateral absence of pupillary and corneal reflexes at 72 h, bilaterally absent N20 somatosensory evoked potential wave at 24 h or later, highly malignant electroencephalography patterns at greater than 24 h, neuron-specific enolase greater than 60 micrograms per liter at 48 and/or 72 h or increasing values, status myoclonus for 30 min or more at or before 72 h, or diffuse anoxic injury on imaging. The middle pathway represents a major 2025 innovation showing good recovery likelihood exceeding 80% when two or more favorable predictors are present with no poor outcome signs: Glasgow Coma Scale motor 4 or 5 at 72–96 h, neuron-specific enolase below 17 micrograms per liter, continuous electroencephalography background without discharges, or absent diffusion restriction on magnetic resonance imaging at days 2–7. The right pathway addresses indeterminate outcome requiring continued observation, repeated assessments, and avoidance of premature withdrawal of life-sustaining therapy. ROSC, return of spontaneous circulation; GCS, Glasgow Coma Scale; NMB, neuromuscular blockade; EEG, electroencephalography; SSEP, somatosensory evoked potentials; NSE, neuron-specific enolase; CT, computed tomography; MRI, magnetic resonance imaging; WLST, withdrawal of life-sustaining therapy.

Indeterminate outcome: When neither concordant unfavorable signs nor favorable signs are present, the neurological outcome remains indeterminate (7, 8). The guidelines suggest observing and re-evaluating patients with indeterminate outcome over time to detect signs of awakening. Although prognosis is generally poor for most of these patients, neurological recovery is still possible (7, 8) (Figure 5).

### Ethical considerations

The 2025 guidelines recommend separating discussions around withdrawal of life-sustaining treatment and the assessment of prognosis for neurological recovery (7, 8). WLST decisions should consider aspects other than brain injury such as age, comorbidity, general organ function, and the patient’s preferences (7, 8).

The guidelines recommend allocating sufficient time for communication around the level-of-treatment decision within the team and with the relatives (7, 8). After a decision on WLST, the guidelines recommend using a structured approach to shift from curative to end-of-life palliative care and considering organ donation (7, 8).

Studies have shown that premature WLST occurring before 72 h for neurological reasons is common and may result in death for patients who might have recovered to good outcome (7, 8). The 2025 guidelines strongly emphasize waiting at least 72 h and excluding all confounders before making prognostic assessments.

### Rehabilitation and long-term follow-up

The 2025 guidelines place significantly enhanced emphasis on rehabilitation and long-term follow-up, recognizing that optimizing neurocritical care extends beyond the acute ICU phase.

### In-hospital rehabilitation

In-hospital rehabilitation should begin as soon as patients are medically stable, including:

- Physical, occupational, and speech therapy tailored to individual needs.
- Active delirium management through non-pharmacological and, if necessary, pharmacological interventions.
- ICU diaries documenting daily events and progress to help patients reconstruct their stay and reduce post-intensive care syndrome incidence.
- Early mobilization as part of comprehensive care (7, 8).

The 2025 guidelines recommend implementing early mobilization, delirium management, and ICU diaries during hospitalization (7, 8).

### Discharge disposition and continuum of care

Not all survivors will be candidates for direct home discharge with 3-month outpatient follow-up. The American Academy of Neurology DoC Guideline (2018) Recommendation #1 emphasizes that patients in states of poor arousal and awareness whose families are not pursuing withdrawal of life-sustaining therapy should be referred to specialized inpatient post-acute rehabilitation facilities to optimize recovery potential (41).

Discharge planning should consider the full continuum of care including:

- Specialized neurorehabilitation units for patients with prolonged DoC.
- Skilled nursing facilities with DoC expertise.
- Acute rehabilitation hospitals for patients regaining consciousness.
- Home-based rehabilitation with outpatient follow-up for functionally independent survivors.

Disposition decisions should be individualized based on level of consciousness, functional status, family preferences, and available regional resources.

### Post-discharge follow-up

Post-discharge follow-up should be organized within 3 months after hospital discharge (7, 8). The guidelines recommend screening for:

- Cognitive deficits including memory problems and executive dysfunction.
- Emotional disturbances including anxiety, depression, and post-traumatic stress disorder (PTSD).
- Physical limitations including fatigue and mobility issues.
- Impact on life roles (7, 8).

A multidisciplinary approach involving neurologists, neuropsychologists, rehabilitation specialists, and social workers is recommended (7, 8). The guidelines recommend performing functional assessments of physical and non-physical impairments before discharge to identify rehabilitation needs and refer to early rehabilitation if indicated (7, 8). Cardiac rehabilitation should be provided as indicated by the cause of the cardiac arrest (7, 8).

### Co-survivor support

Family members and caregivers, termed co-survivors, face significant burden and require:

- Psychological support.
- Education and resources.
- Invitation to follow-up appointments.
- Screening for emotional problems and impact on life roles (7, 8).

The 2025 guidelines recommend inviting co-survivors to the follow-up and asking about emotional problems and impact on life roles (7, 8).

Information should be provided for patients and co-survivors covering:

- Medical subjects including cardiac disease, risk factors, medication, and implantable cardioverter-defibrillator (ICD).
- Potential physical, cognitive, and emotional changes.
- Fatigue, resuming daily activities, driving, work, relationships, and sexuality (7, 8).

### Organ donation

The 2025 guidelines include enhanced recommendations regarding organ donation:

- All patients who have restoration of circulation after cardiopulmonary resuscitation (CPR) and who subsequently progress to death should be evaluated for organ donation (7, 8).
- In comatose ventilated patients who do not fulfill neurological criteria for death, if a decision to start end-of-life care and withdrawal of life support is made, organ donation should be considered when circulatory arrest occurs (7, 8).
- All decisions concerning organ donation must follow local legal and ethical requirements (7, 8).

The prevalence of brain death in ventilated comatose patients with HIBI who died after CPR is 12.6%, with higher prevalence after extracorporeal cardiopulmonary resuscitation (ECPR) at 27.9% versus 8.3% (7, 8). Patients who remain comatose after resuscitation, especially when resuscitated by ECPR, should be actively evaluated for signs of brain death (7, 8). The 2025 guidelines recommend that cardiac arrest registries should report if organ donation after initial resuscitation from cardiac arrest occurred (7, 8).

## Discussion: evolution from 2021 to 2025 ERC/ESICM recommendations

The 2025 ERC/ESICM guidance consolidates a fever-prevention-first strategy, strengthens a pragmatic blood pressure approach, refines EEG and seizure management and multimodal prognostication, and expands the continuum of care to co-survivors and long-term follow-up.

Table 1 presents a comprehensive comparison of key recommendations between the 2021 and 2025 European Resuscitation Council and European Society of Intensive Care Medicine guidelines for post- resuscitation care, highlighting major changes and their clinical implications.

**Table 1:** Key changes from 2021 to 2025 ERC/ESICM post-resuscitation care guidelines.

Domain	2021 recommendations	2025 recommendations	Clinical implications
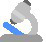 Diagnosis and initial management			
Coronary angiography	Early angiography favored in suspected coronary OHCA	*CHANGED* More selective approach in non-ST-elevation OHCA; delayed unless high clinical suspicion of acute coronary occlusion	Reduce non-beneficial catheterization procedures; target high-yield cases; avoid delays in identifying non-coronary causes
Diagnostic imaging	Selective whole-body CT	*NEW* Strong endorsement of comprehensive CT (head-to-pelvis) when etiology unclear; dual-phase with CTPA if pre-arrest symptoms suggest non-coronary cause	Broader early search for pulmonary embolism, aortic dissection, intracranial hemorrhage; earlier diagnosis enables targeted treatment
Echocardiography	Considered	*STRONG* Routine echocardiography as soon as possible in all patients	Systematic assessment of myocardial dysfunction; guides hemodynamic management and inotrope use
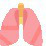 Hemodynamic and respiratory optimization			
Oxygenation target	SpO_2_ 94–98%; avoid hyperoxia	*NEW* SpO_2_ 94–98%; stronger caution against sustained hyperoxia; *NEW*: warning about pulse oximetry overestimating saturation in darker skin tones	Rapid FiO_2_ titration post-ROSC; awareness of pulse oximetry limitations; verify with ABG when available; equity in care
Ventilation target	Normocapnia; PaCO_2_ monitoring	Normocapnia maintained; frequent PaCO_2_ monitoring during hypothermia as hypocapnia may occur	Proactive ABG monitoring; avoid cerebral vasoconstriction from hypocapnia
Blood pressure target	MAP ≥65 mmHg; individualization discussed but not strongly defined	*CHANGED* MAP 60–65 mmHg as default; explicit individualization criteria (chronic hypertension or persistent organ hypoperfusion)	Shift from universal higher MAP to personalized moderate-intensity targets; spare vasopressor burden in many patients; reduce complication
Vasopressor selection	Noradrenaline generally preferred	*NEW* Noradrenaline first-line; adrenaline acceptable when noradrenaline unavailable (e.g., prehospital)	Pragmatic approach for resource-limited settings; maintains safety while acknowledging real-world constraints
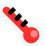 Temperature management			
Temperature management	TTM at 33 °C or 36 °C for 24 h; both options acceptable	*PARADIGM SHIFT* Universal fever prevention ≤37.5 °C for 36–72 h; no routine hypothermia; term changed to temperature control	Paradigm shift from hypothermia induction to fever prevention alone; simplified protocols; reduced shivering; less physiological stress
Prehospital cooling	Not specifically addressed	*STRONG* Recommend AGAINST routine cold fluid infusion (strong recommendation)	Avoid potential harm from prehospital cooling; focus resources on post-ROSC stabilization
Rewarming mild hypothermia	Not specifically addressed	*NEW* Do NOT actively rewarm patients with mild hypothermia (32–36 °C) after ROSC	Allow spontaneous rewarming; avoid additional physiological stress
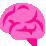 Neurological management			
Seizure prophylaxis	Noncommittal on prophylaxis	*CHANGED* Explicitly AGAINST routine prophylaxis (weak recommendation)	Avoid unnecessary medication side effects, sedation, and drug interactions; focus on treating confirmed seizures only
EEG timing	Continuous EEG encouraged for 24–48 h	*NEW* EEG from day 1 after ROSC; record during myoclonic jerks	Earlier prognostic data; better seizure detection; identify background patterns suggesting recovery potential
EEG for myoclonus	General recommendation to treat myoclonus	Record EEG during myoclonic jerks; attempt wake-up trial if myoclonus with benign EEG	Distinguish epileptiform from non-epileptiform myoclonus; avoid over-treatment; identify patients with recovery potential
Sedation strategy	Short-acting agents recommended	*STRONG* Strong emphasis on daily sedation holds and early neurological examination	Enable earlier prognostic clarity; potentially shorter ICU stays; facilitate wake-up assessment
Neuromuscular blockade	Selective use when indicated	*AGAINST* Explicitly AGAINST routine use; only for severe ARDS or refractory status epilepticus or shivering	Minimize risks of prolonged paralysis; allow clinical examination; enable seizure detection
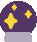 Neurological prognostication			
Prognostication threshold	GCS motor ≤3 at ≥72 h	*EXPANDED* GCS motor <6 at ≥72 h	Broader eligibility for prognostication; capture more patients requiring assessment; earlier family counseling
Pupil assessment	Clinical examination of pupillary light reflex	*NEW* Recommend pupillometry when available	Standardized objective measurement; reduce inter-rater variability; improve prognostic accuracy
Corneal reflex	Part of clinical examination	Bilateral absence at ≥72 h for poor outcome prediction (weak recommendation)	Continued use in multimodal assessment; requires careful assessment technique
SSEP timing	Generally recommended ≥72 h	*CHANGED* Acceptable from ≥24 h (changed from previous)	Earlier prognostic information; faster decision-making capability; still requires NMB during test
EEG reactivity	Considered poor outcome predictor	*MAJOR CHANGE* AGAINST using absence alone to predict poor outcome (weak recommendation)	Reduced false-positive predictions; requires integration with other modalities; major change from previous practice
Status myoclonus definition	Variable definitions	*STANDARDIZED* Defined as continuous generalized myoclonus for ≥30 min within 72 h	Standardized definition; improved consistency across centers; part of multimodal assessment
NSE thresholds	>60 μg/L at 48-72 h	>60 μg/L at 48 and/or 72 h; OR increasing NSE between timepoints; serial sampling at 24–48-72 h	Detect trends; minimize hemolysis confounding; improved sensitivity
Other biomarkers	S-100B mentioned	*AGAINST* Do NOT recommend NfL; S-100B; GFAP; tau (absence of consistent thresholds)	Focus on validated biomarkers (NSE); avoid premature clinical use of research markers
Brain CT findings	General recommendation for imaging	Generalized edema (reduced grey matter to white matter ratio) to predict poor outcome; repeat CT at 72-96 h if initial normal but coma persists	Specific imaging criteria; proactive re-imaging strategy; detect evolving injury
Brain MRI	Mentioned as useful	Extensive diffusion restriction at 2–7 days predicts poor outcome	Specific timing and pattern criteria; useful when CT inconclusive
Imaging expertise	General recommendation	*NEW* Emphasize evaluation by specialist with specific experience; consider telemedicine consultation where unavailable	Quality assurance for prognostication; reduce interpretation errors; expand access via telemedicine
Favorable predictors	Not systematically defined	*NEW* Formal favorable predictors introduced: GCS motor 4–5 at 72-96 h; NSE < 17 μg/L; continuous EEG; no DWI restriction on MRI	Balanced prediction of both poor and good outcomes; >80% recovery rate with ≥2 favorable signs; realistic family counseling
Indeterminate outcome	Not formally recognized	*NEW* Explicit indeterminate category when neither poor nor favorable signs predominate; recommend prolonged observation	Avoid premature WLST in uncertain cases; acknowledge prognostic uncertainty; define management pathway
Prognostication algorithm	Required ≥2 poor predictors	*STRENGTHENED* Requires ≥2 concordant predictors from multiple domains; can be poor OR favorable	Multimodal approach strengthened; recognize spectrum of outcomes; reduce errors
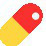 Supportive care			
Prophylactic antibiotics	Standard ICU practice	*AGAINST* Recommend AGAINST routine use; low threshold for empiric treatment with clinical suspicion of pneumonia	Antibiotic stewardship; balance with high aspiration risk post-arrest; individualized approach
Stress ulcer prophylaxis	General ICU recommendation	*STRONG* Emphasized given high GI bleeding risk with antiplatelet/anticoagulant therapy	Proactive GI protection; recognize increased bleeding risk
Nutrition	Early enteral feeding	Start trophic feeding, increase gradually as tolerated	Early gut nutrition; avoid feeding intolerance; support metabolic recovery
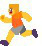 Rehabilitation and follow-up			
In-hospital rehabilitation	Mentioned briefly	*COMPREHENSIVE* Comprehensive guidance: early mobilization, delirium management, ICU diaries, physical/occupational/speech therapy	Systematic implementation; reduce post-intensive care syndrome; improve functional outcomes
Post-discharge follow-up	General recommendation	*MANDATORY* Mandatory within 3 months; structured screening for cognitive/emotional/physical impairments; multidisciplinary teams	Proactive long-term care; early detection of problems; coordinated intervention
co-survivor support	Not specifically addressed	*NEW* Formal recognition of co-survivors; psychological support; invitation to follow-up; screening for emotional problems	Address caregiver burden systematically; include family in recovery; prevent co-survivor distress
Patient information	General recommendation	*SPECIFIC* Specific information covering medical topics, resuming activities (driving, work, relationships, sexuality)	Comprehensive patient education; empower decision-making; facilitate return to normal life
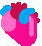 Organ donation and registry reporting			
Organ donation	Consider in appropriate circumstances	*MANDATORY* Mandatory evaluation of all post-arrest deaths; highlight 12.6% brain death overall and 27.9% after ECPR; active assessment in ECPR survivors	Systematic vs. opportunistic approach; maximize organ utilization; recognize ECPR as high brain death risk
Registry reporting	Report standard outcomes	*NEW* Registries should report organ donation after cardiac arrest	Improve data on donation rates; enable quality improvement

Comprehensive comparison of key recommendations between 2021 and 2025 European Resuscitation Council and European Society of Intensive Care Medicine guidelines for post-resuscitation care.

TTM, targeted temperature management; SpO_2_, peripheral oxygen saturation; FiO_2_, fraction of inspired oxygen; ABG, arterial blood gas; MAP, mean arterial pressure; mmHg, millimeters of mercury; GCS, Glasgow Coma Scale; EEG, electroencephalography; SSEP, somatosensory evoked potentials; NMB, neuromuscular blockade; NSE, neuron-specific enolase; NfL, neurofilament light chain; GFAP, glial fibrillary acidic protein; DWI, diffusion-weighted imaging; MRI, magnetic resonance imaging; CT, computed tomography; GM/WM, grey matter/white matter; WLST, withdrawal of life-sustaining therapy; ICU, intensive care unit; ARDS, acute respiratory distress syndrome; GI, gastrointestinal; ECPR, extracorporeal cardiopulmonary resuscitation; OHCA, out-of-hospital cardiac arrest; CTPA, computed tomography pulmonary angiography; ROSC, return of spontaneous circulation; ICP, intracranial pressure; μg/L, micrograms per liter; kPa, kilopascals.

### Initial diagnostics and early management

The 2021 guidelines favored early coronary angiography in OHCA with suspected coronary cause and considered whole-body CT selectively. The 2025 guidelines recommend more selective invasive cardiology in non-ST-elevation OHCA, encouraging delayed or targeted angiography unless high suspicion exists, provide stronger endorsement of early comprehensive CT from head to pelvis when etiology is unclear, and show greater attention to post-ROSC myocardial dysfunction surveillance through routine echocardiography (7, 8). The clinical implication is to reduce non-beneficial early catheterization in non-ST-elevation patterns, broaden early search for non-coronary etiologies, and embed point-of-care echocardiography in the first hours.

### Oxygenation and ventilation

The 2021 guidelines recommended avoiding hypoxia and hyperoxia, targeting SpO_2_ of 94–98%, and maintaining normocapnia. The 2025 guidelines reaffirm narrow oxygen targets with stronger caution against sustained hyperoxia, reinforce lung-protective ventilation and strict normocapnia, and provide a new warning about pulse oximetry overestimating oxygen saturation in darker skin tones (7, 8). The clinical implication is to titrate fraction of inspired oxygen quickly post-ROSC, proactively monitor arterial blood gases, be aware of pulse oximetry limitations, and avoid both hypocapnia and hypercapnia.

### Hemodynamics and blood pressure targets

The 2021 guidelines generally recommended MAP at or above 65 mmHg with individualized targets discussed but not strongly qualified. The 2025 guidelines endorse MAP of 60–65 mmHg as default, based on individual patient data meta-analysis showing no global benefit of higher MAP greater than 71–85 mmHg, with more explicit criteria for individualization in chronic hypertension or organ hypoperfusion and acceptance of prehospital adrenaline infusions when noradrenaline unavailable (7, 8). The clinical implication is a shift from universal higher MAP to personalized moderate-intensity targets, sparing vasopressor burden in many patients while recognizing scenarios requiring higher MAP.

### Temperature control

The 2021 guidelines recommended temperature control with either 33 or 36 degrees Celsius, acknowledging the TTM trial showing no difference between these targets. The 2025 guidelines pivot to universal fever prevention targeting temperature at or below 37.5 degrees Celsius for 36–72 h without routine hypothermia, rename the approach as temperature control versus targeted temperature management, and clearly advise against routine prehospital cooling with cold fluids (7, 8). The clinical implication represents a paradigm shift from active hypothermia induction to active fever prevention alone, simplifying protocols, reducing shivering burden, and minimizing physiological stress while retaining key neuroprotection. The TTM2 trial and subsequent analyses support equivalence of 33 versus normal temperatures with active fever prevention, and fever itself remains detrimental.

### Seizure management

The 2021 guidelines encouraged continuous EEG for seizure detection and treated overt seizures with first- line levetiracetam or sodium valproate but were noncommittal on prophylaxis. The 2025 guidelines explicitly recommend against routine prophylactic antiseizure drugs, advocate treating only confirmed seizures on clinical examination or EEG, promote early EEG from day 1 to detect both seizures and prognostic markers such as background continuity and reactivity, emphasize wake-up trials in patients with myoclonus plus benign EEG, and recommend recording EEG during myoclonic jerks to identify any epileptiform correlate (7, 8). The clinical implication is to avoid medication side effects and sedation from unnecessary prophylaxis, focus treatment on proven seizures, and maximize early prognostic data from EEG while enabling earlier awakening trials.

### Prognostication

The 2021 guidelines recommended multimodal prognostication at 72 h or later, combining clinical examination including pupillary and corneal reflexes, bilateral absence of N20 on SSEPs, specific malignant EEG patterns, NSE greater than 60 micrograms per liter at 48–72 h, status myoclonus, and imaging findings including CT or MRI. The 2025 guidelines lower the awakening threshold for prognostication from Glasgow Coma Scale motor less than or equal to 3 to motor less than 6, strongly recommend pupillometry for objective pupil assessment, downgrade EEG reactivity as a standalone poor-outcome predictor, clarify SSEP timing as acceptable from 24 h rather than requiring delay until 72 h, refine status myoclonus definition as continuous generalized myoclonus for 30 min or more within 72 h, introduce formal favorable predictors including Glasgow Coma Scale motor 4–5 at 72–96 h, NSE less than 17 micrograms per liter, continuous EEG background without discharges, and absence of diffusion restriction on MRI, emphasize indeterminate outcome category when neither poor nor favorable signs predominate, and recommend repeat imaging at 72–96 h if initial CT is normal but coma persists (7, 8). The clinical implication is broader eligibility for prognostication capturing more patients, standardized pupil measurement reducing inter-rater variability, earlier SSEP feasibility, balanced prediction of both poor and good outcomes to guide realistic family counseling, and defined pathway for uncertain cases including re- imaging and prolonged observation rather than premature withdrawal.

### Sedation and neuromuscular blockade

The 2021 guidelines recommended short-acting sedation to enable neurological assessment and selective neuromuscular blockade only when indicated. The 2025 guidelines strengthen recommendations for daily sedation holds and early neurological examination, explicitly state against routine neuromuscular blockade as it obscures seizures and clinical signs, and restrict paralysis to severe ARDS, refractory status epilepticus, or shivering during active cooling (7, 8). The clinical implication is greater emphasis on wake-up trials enabling earlier prognostic clarity and potentially shorter ICU stays while minimizing risks of prolonged paralysis.

### General care

The 2021 guidelines recommended standard ICU supportive care including glycemic control, nutrition, and infection prevention. The 2025 guidelines add explicit recommendations against routine prophylactic antibiotics, favor low threshold for empiric antibiotics with clinical suspicion of pneumonia particularly given aspiration risk, recommend early trophic enteral feeding with gradual advancement, and emphasize stress ulcer prophylaxis given high gastrointestinal bleeding risk with antiplatelet or anticoagulation therapy (7, 8). The clinical implication is antibiotic stewardship balanced with vigilance for post-arrest aspiration pneumonia, early gut nutrition supporting metabolic recovery, and proactive gastrointestinal protection.

### Rehabilitation and long-term care

The 2021 guidelines mentioned rehabilitation and follow-up briefly without detailed recommendations. The 2025 guidelines introduce comprehensive guidance for in-hospital early mobilization, delirium management, and ICU diaries, mandate structured follow-up within 3 months post-discharge screening for cognitive, emotional, and physical impairments, recommend multidisciplinary rehabilitation teams, formally recognize co-survivors requiring psychological support and inclusion in follow-up, and specify providing detailed information on resuming activities including driving, work, and relationships (7, 8). The clinical implication represents a major expansion of post-ICU care continuum, addressing post-intensive care syndrome, supporting caregiver burden, and optimizing functional recovery and quality of life beyond hospital survival.

### Organ donation

The 2021 guidelines mentioned organ donation consideration in appropriate circumstances. The 2025 guidelines mandate evaluation of all post-cardiac arrest deaths for donation eligibility, highlight 12.6% brain death prevalence overall and 27.9% after ECPR, recommend active brain death assessment in comatose ECPR survivors, suggest considering donation after circulatory determination of death when life- sustaining treatment is withdrawn, and require cardiac arrest registries to report donation outcomes (7, 8). The clinical implication is systematic rather than opportunistic donation evaluation, recognition of ECPR as high brain death risk warranting proactive assessment, and improved organ utilization from cardiac arrest deaths.

## Conclusion

The 2025 ERC/ESICM guidelines for post-resuscitation care represent a significant evolution in the management of comatose cardiac arrest survivors, emphasizing evidence-based neuroprotection through rigorous fever prevention rather than routine hypothermia, individualized hemodynamic management targeting moderate mean arterial pressure of 60–65 mmHg, balanced oxygenation with awareness of pulse oximetry limitations, and selective rather than prophylactic antiseizure medication use. The refined multimodal prognostication algorithm incorporates both poor and favorable outcome predictors, requires assessment at 72 h or later with exclusion of all confounders, mandates concordance of at least two predictors to minimize false-positive predictions of poor outcome, and introduces formal recognition of indeterminate outcomes requiring prolonged observation. Perhaps most notably, the 2025 guidelines expand the continuum of care beyond the ICU phase, establishing comprehensive rehabilitation pathways, structured 3-month follow-up assessments for both survivors and co-survivors, and systematic evaluation for organ donation in all appropriate cases. Implementation of this integrated multimodal framework offers clinicians the optimal evidence-based approach to maximize neurological recovery, provide accurate prognostic information to families, avoid premature withdrawal of life-sustaining therapy, and improve long-term functional outcomes and quality of life for cardiac arrest survivors.
